# Detection and classification of adult epilepsy using hybrid deep learning approach

**DOI:** 10.1038/s41598-023-44763-7

**Published:** 2023-10-16

**Authors:** Saravanan Srinivasan, Sundaranarayana Dayalane, Sandeep kumar Mathivanan, Hariharan Rajadurai, Prabhu Jayagopal, Gemmachis Teshite Dalu

**Affiliations:** 1 Department of Computer Science and Engineering, Vel Tech Rangarajan Dr. Sagunthala R&D Institute of Science and Technology, Chennai, 600062 India; 2 School of Computing Science and Engineering, Galgotias University, Greater Noida, 203201 Uttar Pradesh India; 3 School of Computing Science and Engineering, VIT Bhopal University, Bhopal–Indore Highway Kothrikalan, Sehore , 466114 Madhya Pradesh India; 4 School of Computer Science Engineering and Information Systems, Vellore Institute of Technology, Vellore, 632014, Tamil Nadu India; 5 Department of Software Engineering, College of Computing and Informatics, Haramaya University, POB 138, Dire Dawa, Ethiopia

**Keywords:** Diseases, Health care, Medical research

## Abstract

The electroencephalogram (EEG) has emerged over the past few decades as one of the key tools used by clinicians to detect seizures and other neurological abnormalities of the human brain. The proper diagnosis of epilepsy is crucial due to its distinctive nature and the subsequent negative effects of epileptic seizures on patients. The classification of minimally pre-processed, raw multichannel EEG signal recordings is the foundation of this article’s unique method for identifying seizures in pre-adult patients. The new method makes use of the automatic feature learning capabilities of a three-dimensional deep convolution auto-encoder (3D-DCAE) associated with a neural network-based classifier to build an integrated framework that endures training in a supervised manner to attain the highest level of classification precision among brain state signals, both ictal and interictal. A pair of models were created and evaluated for testing and assessing our method, utilizing three distinct EEG data section lengths, and a tenfold cross-validation procedure. Based on five evaluation criteria, the labelled hybrid convolutional auto-encoder (LHCAE) model, which utilizes a classifier based on bidirectional long short-term memory (Bi-LSTM) and an EEG segment length of 4 s, had the best efficiency. This proposed model has 99.08 ± 0.54% accuracy, 99.21 ± 0.50% sensitivity, 99.11 ± 0.57% specificity, 99.09 ± 0.55% precision, and an F1-score of 99.16 ± 0.58%, according to the publicly available Children’s Hospital Boston (CHB) dataset. Based on the obtained outcomes, the proposed seizure classification model outperforms the other state-of-the-art method’s performance in the same dataset.

## Introduction

**Figure 1:**
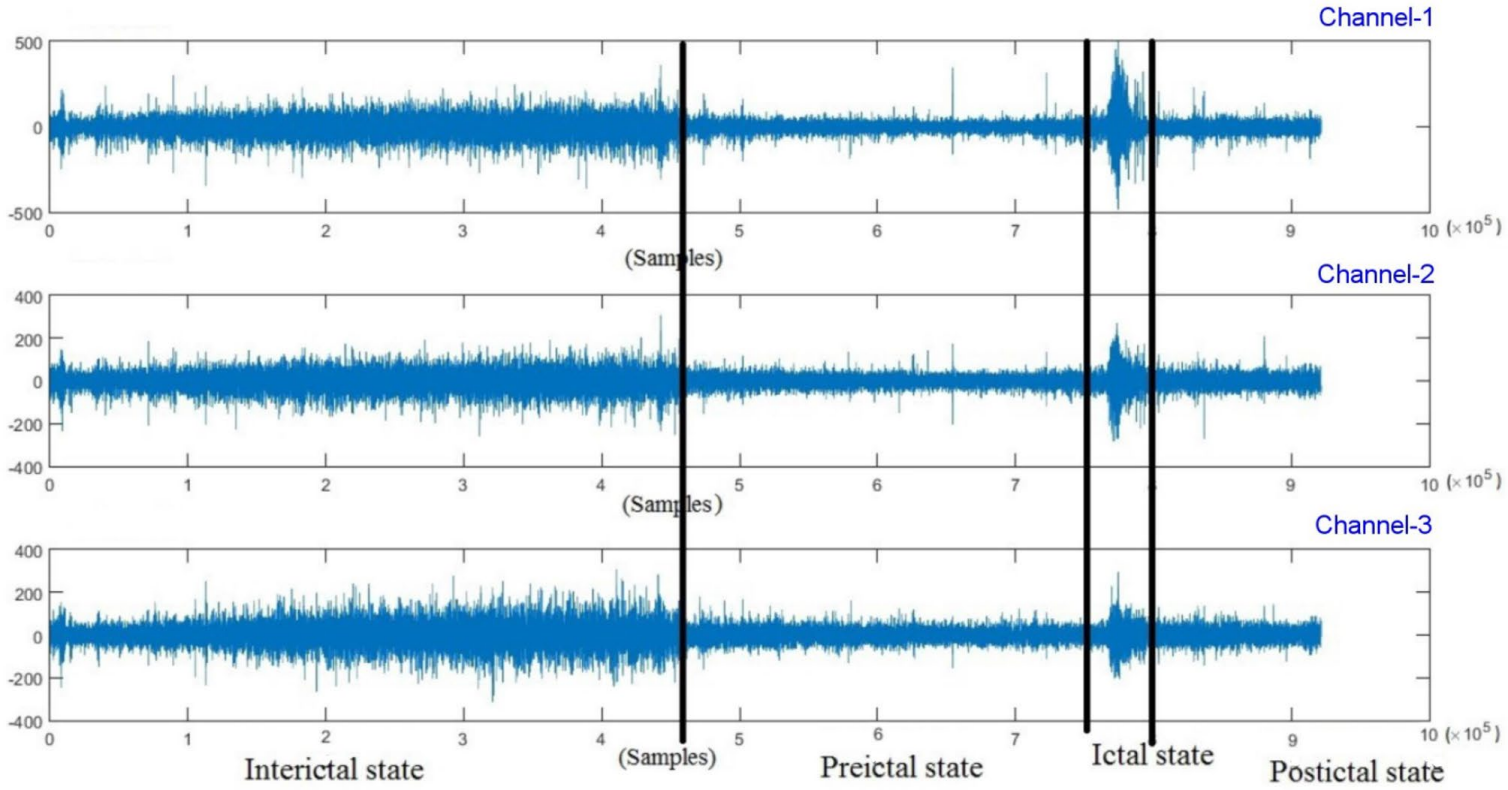
Seizure states like interictal, preictal, ictal, and postictal from three channels, each of which was recorded for an hour.

Seizures are a common symptom of epilepsy, a neurological disorder. This disease affects more than 1% of the world’s population. Medical treatment and surgical treatment are available for patients suffering from this disease. More than 30% of patients with seizures who develop subsequent seizures are unable to control them with medication or surgical procedures, even when seizures have occurred^1^. The importance of predicting subsequent seizures is therefore extremely important in order to be able to prevent them with medication before they occur. A brain’s electrical activity can be monitored by recording electroencephalogram (EEG) signals. Patients’ scalp EEGs or intracranial EEGs (iEEGs) signals can be recorded using electrodes inserted into their brain tissues or placed on their scalps. When the brain has neurological issues, the electrical signals inside can suddenly change. This change shows up in EEG readings^2^. The Fig. 1 shows three lines representing 1 h of brain wave activity recorded from EEG signal. The lines cover three conditions: Pre-seizure condition: the 30 min before a seizure happens. During-seizure condition: the beginning and end of the actual seizure. Post-seizure condition: The period right after a seizure. The time before a seizure occurs can provide valuable clues^3^. Preictal state refers to the period right before a seizure takes place. It can offer information about what triggers a person’s seizures and how soon they may happen. Epileptic seizures and brain disorders can be identified using EEG signals. EEG tests are non-invasive, have good time resolution, are low cost, and are safe. Epileptic seizures happen when groups of brain cells suddenly send abnormal signals^4^. This causes temporary changes in how the brain works. Sometimes seizures go unnoticed or can be mixed up with other brain conditions like meningitis or stroke that cause similar symptoms. EEG signals are useful for identifying many health problems including schizophrenia, Alzheimer’s disease, sleep issues, seizures, brain tumors, and infections of the brain and nervous system^5^.

The passage talks about detecting epilepsy from brain wave signals. It says precise analysis of brain waves from people with epilepsy can provide useful facts. The brain waves are complex, so analyzing them needs looking at many factors. Doctors checking brain waves themselves has helped spot patterns. But this requires high skills and knowing many analysis tools. Recently, automating how computers detect epilepsy a seizure has gotten researchers interested^6^. The text talks about how EEG signals are analyzed. EEG signals measure electrical activity in the brain. Over time, technology has improved to allow digital analysis of EEG data. This helps detect seizures in the brain. The digital EEG analysis has three main steps: pre-processing, feature extraction, and classification. Pre-processing involves preparing the raw data by removing noise and enhancing the signal. Feature extraction identifies important characteristics in the data. This helps identify patterns related to seizures. Some features may be removed during optional feature selection to improve the analysis. Classification assigns the data to different groups. The classification model looks for patterns in the features to identify seizures. Overall, digital EEG analysis uses signal processing and machine learning techniques to detect seizures and other brain conditions from EEG data. The text describes the general workflow of pre-processing, feature extraction and classification^7^. The proposed framework uses a 3D deep convolution auto-encoder (3D-DCAE) to detect epileptic seizures from EEG recordings. The auto-encoder (AE) is trained once in a supervised way to do two things at the same time. First, it learns the best features from the EEG signals. It then summarizes them into a simple, low-dimensional representation. This helps it classify the signals efficiently. Training the auto-encoder to do both tasks at once has proven very helpful. It improves how well the model learns. This leads to better accuracy when classifying the EEG signals. The advantages of our approach are as follows: First, our LHCAE model trains faster than standard supervised methods since it trains only once. Second, to limit parameters, we use convolutional layers instead of fully connected layers. This helps the model learn features from the EEG data. Third, our system can compress the original high-dimensional signals using the low-dimensional latent representations from the decoder part. Fourth, training autoencoder in a supervised way helps learn structured latent representations. This allows us to use simple classifiers with high precision for seizure detection. Finally, we considered performance and hardware resources to make our system suitable for real-time use and hardware implementation. This can help with deployment as well. The two models aim to detect seizures in minors. They both differentiate between ictal and interictal brain states by classifying EEG data. A combination of simple multi-layer perceptron and hooked-up 3D convolutional layers helps classify data in Model 1. 3D convolutional layers are also utilized by Model 2. For performing the classification task, their attachment with a Bi-LSTM layer is necessary. We evaluate these models’ efficacy by comparing their performance to two other standard deep learning algorithms with identical architectures. The decoder layers were taken out. Supervised training has restricted the use of these models to just classification tasks. Our approach of utilizing LHCAE shows promise for precise seizure detection by experimenting with various EEG segment lengths.

## Related work

Detecting seizures early presents a critical obstacle in the management of epilepsy. Numerous scientists have attempted to discover methods for precisely predicting seizure onset. A seizure prediction mechanism that utilizes deep learning methods is suggested by the research. To execute this method, first pre-process the scalp EEG signals. Then extract features by employing a convolutional neural network followed by classification of the data via support vector machines. The method was successful in achieving an average sensitivity of 92.7% and specificity of 90.8%, as demonstrated by testing on data from 24 subjects^8^. Intracranial EEG data from 10 patients was analyzed as part of a pseudo prospective seizure prediction study in another research effort. A deep learning classifier was trained initially to distinguish preictal and interictal signals Afterward, the classifier’s performance was evaluated on EEG data that were held out from all patients and compared to a random predictor. The prediction system may be calibrated to prioritize sensitivity or time in alerting, depending on the needs of the patient. The system’s prediction achieved a mean sensitivity of 69% and time in warning of 27%, surpassing a random predictor’s performance by 42% for all patients. An ultra-low power neuromorphic chip for wearables to run the prediction system was demonstrated as feasible by the researchers^9^. The model suggested was designed to enhance the quality and simplify technical terms. Two different algorithms were employed to test the model: K-nearest neighbours (KNN) and Support Vector Machines (SVM). Both algorithms produced comparable outcomes. SVM and KNN have shown that our method of extracting features using feature engineering techniques in the three different domains was highly accurate in its measurements of specificity and sensitivity. The contribution of t-test and sequential forward feature selection cannot be underestimated in the success achieved. With just five features it’s possible to create a trustworthy model that achieves excellent results; SVM delivered perfect scores for accuracy, sensitivity and specificity while KNN scored an impressive overall result of more than 99%. Our approach is effective in achieving high accuracy using minimal features^10^. Differentiating between epileptic seizures through varying characteristics was the main thrust behind reviewing literature on feature selection. They put together a classification of standard solutions used when dealing with this problem, while the main focus of this analysis was on how classifiers operate on distinct open-source datasets. In their conclusion regarding predictive methods for epilepsy the research identified opportunities along with potential gaps and challenges^11^. Epilepsy results in recurrent and sudden seizures, and anticipating seizure activity sooner can significantly enhance patients’ health outcomes. After many years of studying the problem remains: how to predict seizures? The cause of this issue may be due to limited data accessibility. However, exciting new machine learning (ML) based tools give us a better chance at predicting seizures early and accurately. Using EEG signals and machine learning methods to detect seizures as early as possible^12^.

Different machine learning methods were applied to examine information about epileptic seizures. When compared with other algorithms like K nearest neighbours, naive bayes, logistic regression, decision tree, random tree and J48; the random forest model stood out for its superior performance. Stochastic gradient descent underwent testing as well. With an accuracy level of 97.8%, a ROC score at 0.96, and root mean squared error at 0.527, the random forest model performed very well. Additional examination was conducted on several models to evaluate their ability to classify the epileptic seizure data. Their parameters underwent minor changes^13^. The HVD single elements’ instantaneous amplitude is affected by movement artifacts in brainwave signals. The identification of epileptic seizures and normal human activities is aided by analyzing the statistical features acquired through instantaneous amplitude. To choose the features, we rely on a Q-score based on correlation, and subsequently classify them through an LSTM model in deep learning. Maximizing the accuracy is achieved through feature-based weight update. Our Sensor Networks Research Lab data was utilized for epilepsy diagnosis using the Bonn dataset, and our proposed method achieved test classification accuracies of 96.0% through activity recognition. A test classification accuracy of 83.0% is achieved by the proposed method for recognizing activities^14^.

The proposed approach was tested on numerous genuine EEG readings. Sensitivity, specificity and accuracy are the criteria for evaluating performance. Different scenarios are utilized to run various experiments, such as healthy individuals with eyes open or closed. When there are no seizures originating from two specific areas of the brain, patients with epilepsy experience this. Additional scenarios are tested with extra background noise generated by physical and environmental elements. The identification of seizure and non-seizure segments shows great results with an excellent performance. It shows great resilience against sources of noise^15^. A seizure detection and prediction method were attempted to be created by researchers using the stacked bidirectional long short-term memory technique. Analyzing time series data using this method solves the vanishing gradient problem in recurrent neural networks. For the detection and prediction experiments, data from Bonn University was gathered. The seizure detection accuracy of our model was the highest, reaching 99.08%, with precision at 98%, recall at 99.5% and an ROC AUC score of 0.84346. Exceeding 90%, an accuracy in a binary classification is considered outstanding. By identifying preictal states of EEG readings from both interictal and ictal states, seizure prediction was performed using the same data^16^. Data mining methods are applied to EEG signal analysis in order to detect seizures automatically based on the information presented in this passage. To extract features from time series data, the developers designed a versatile tool called Training Builder. By using signal processing techniques combined with a sliding window approach and both feature extraction and selection methods alongside Support Vector Machines the trained classifier was able to operate successfully. The seizure detection accuracy of the model was over 99% during testing on public EEG datasets, demonstrating outstanding results^17^. The new method for seizure detection using Stein kernel-based sparse representation (SR) is introduced in the paper for EEG recordings. The construction of SR within the SPD matrix space is done by this framework, unlike traditional methods that operate with data within a flat-space. A space that curves is formed by these matrices. Curved geometries are embedded in a high-dimensional feature space using the Stein kernel to perform SR. This framework represents EEG samples as CovDs. The representation of the test sample using training data in a sparse way is followed by classification into a category that exhibits minimum discrepancy between its initial and restored version^18^. The content discusses comparing different ways to extract features from EEG seizure data with the goal of detecting seizures. Both direct extraction of features from the original signals and using adaptive decomposition methods were evaluated. The outcomes of the various decomposition techniques evaluated were relatively similar overall, but VMD and CEEMDAN yielded somewhat better results. Inferior class separability was observed when extracting features solely from the original signals without any decomposition. Nevertheless, certain classifiers enabled accurate predictions. By presenting a standardized methodology, this study enables more accurate comparisons between different EEG seizure detection methods. We employed the same classifiers, parameters, spectral features in both time domain^19^. The investigators established a technique to classify seizures that operates rapidly and dependably. They merged deep neural networks with higher-order statistics. This method involves extracting key structural features from third-order cumulant coefficient matrices using a sparse autoencoder neural network. The softmax classifier can effectively categorize EEG signals into two or three groups with high accuracy. The scientists conducted an experiment using EEG data that was publicly available from the University of Bonn^20^.

The study aims to use MFCCs for classification purposes after computing them during the feature extraction phase. A filter bank with bandwidths close to the critical bandwidths of the human ear is used in calculating MFCCs through frequency analysis. Most prior research employing this identical data set have been outperformed by our suggested approach concerning classification accuracy, sensitivity and specificity. Extracting features using cepstral analysis was instrumental in achieving this success. Real-time seizure detection systems can benefit from the potential of this system. The inherent nature of the proposed method’s neural network is non-iterative^21^. Author employed a two-step process in our methodology. Initially, we utilized labelled multi-lead EEG short samples to train squeeze-and-excitation networks (SENet), aiming to extract short-term features. Concurrently, we trained long short-term memory networks (LSTM) using compressed data, focusing on extracting long-term characteristics and constructing a classifier. During the inference phase, we introduced an additional step. Initially, we adjusted the LSTM feature mapping using adversarial learning. This process involved a dynamic interaction between the LSTM and the clustering subnet. Its purpose was to align the EEG data from the target patient with the EEG data in the database, ensuring that they follow the same distribution within the deep feature space. In the concluding step, the adapted classifier is deployed to ascertain the specific type of seizure. Our experiments encompassed the utilization of both the TUH EEG Seizure Corpus and the CHB-MIT seizure database. The empirical results unequivocally demonstrate that the proposed domain-adaptive deep feature representation significantly enhances the classification accuracy of the hybrid deep model within the target dataset, yielding an impressive 5% improvement^28^. The author harnessed the automatic feature learning capabilities of a two-dimensional deep convolution autoencoder (2D-DCAE) integrated with a neural network-based classifier. This integration formed a unified system that underwent supervised training to achieve optimal classification accuracy between ictal and interictal brain state signals. In the pursuit of evaluating our technique, we constructed and tested two distinct models. These models were assessed using three different EEG data segment lengths and a rigorous tenfold cross-validation scheme. After comprehensive evaluation based on five assessment criteria, the highest-performing model emerged as the supervised deep convolutional autoencoder (SDCAE) model. This model featured a bidirectional long short-term memory (Bi-LSTM)—based classifier and employed EEG segment lengths of 4 s^29^. The aim of this endeavour is to develop hardware-implementable machine learning classifiers capable of accurately predicting seizure onsets with high sensitivity. The proposed classification methodology involves a multi-step process, which includes channel selection tailored to each patient, feature extraction from EEG data, the identification of the optimal feature combination for each patient, and subsequent training of the selected support vector machine (SVM) classifier. Upon evaluating the performance of this classification approach, the results are highly encouraging. In several cases, the achieved accuracy exceeds 95%, signifying the robustness and effectiveness of the proposed methodology^30^.

This paper presents a novel automated seizure-detection technique that provides users with three distinct tactics, allowing them to choose the most suitable one for a specific categorization task. Notably, the feature extraction process encompasses both linear and nonlinear measures, extracted directly from the EEG signals. Additionally, features can be derived from the sub-bands of the tunable-Q wavelet transform (TQWT) or even from the intrinsic mode functions (IMFs) obtained through multivariate empirical mode decomposition (MEMD). The classification task is carried out efficiently using a support vector machine (SVM). Author evaluated the performance of our proposed method using a publicly available database, considering six binary classification scenarios designed to distinguish between healthy, seizure, and non-seizure EEG signals. In comparison to state-of-the-art techniques, our results showcase superior accuracy (ACC), sensitivity (SEN), and specificity (SPE)^31^. In this article, we employ the Superlet Transform (SLT) in conjunction with a deep convolutional neural network, specifically VGG-19, for the purpose of detecting both seizure and non-seizure events. Our proposed approach is rigorously validated using an electroencephalogram (EEG) dataset sourced from the University of Bonn. Remarkably, our suggested technique exhibits exceptional performance, achieving a perfect 100% accuracy across all seven instances of seizure and non-seizure detection studied in this research. Notably, it outperforms other established methods when dealing with three and five-class classification challenges. Furthermore, to further assess the robustness of our technique, we applied it to the CHB-MIT scalp EEG database. In this context, our proposed method demonstrated a remarkable classification accuracy of 94.3% in effectively discriminating between seizure and non-seizure episodes^32^. This paper introduces a novel hybrid technique that combines higher-order statistics (HOS) with sensitivity analysis and the residual wavelet transform (RWT). The sensitivity analysis approach focuses on specific segments of the brain signal primarily influenced by transient and burst events, measuring a set of frequencies associated with underlying nonlinear dynamics. Leveraging these frequency standards, the proposed method assesses brain output across two distinct regions. Additionally, the RWT is employed to analyze non-stationary time series in time-scale space, effectively detecting transient and impulsive changes. The results demonstrate the effectiveness of this approach, achieving an impressive discriminating accuracy of 99.76% on the Bern Barcelona EEG database^33^. The proposed network is constructed upon an autoencoder, delving deep into the exploration of the intricate non-linear dynamics inherent in electroencephalogram (EEG) inputs. This approach leverages established deep neural domain expertise to distil valuable insights from raw data, ultimately culminating in the development of a sophisticated deep neural network-based learning model. This model is designed to effectively predict the nature of unknown seizures. Within this framework, EEG waves are channelled into a neural network founded on autoencoder principles. Notably, this network inadvertently uncovers and harnesses pertinent properties that prove instrumental in the subsequent operation of the softmax classifier^34^. The objective of this chapter is to delve into the analysis of brain activity dynamics derived from electroencephalogram (EEG) signals, with a specific focus on identifying the seizures associated with epilepsy. Consequently, the primary aim of this article is to perform robust seizure classification. In pursuit of this goal, we propose a nonlinear higher-order spectral approach in this paper to scrutinize the intricate dynamics inherent in nonstationary EEG data. From the prominent realm of higher-order spectra, a diverse array of statistical characteristics is meticulously extracted. This extraction process is executed through the data reduction technique known as location-sensitive discriminant analysis (LSDA)^35^.

## Materials and methods

### Materials

**Figure 2:**
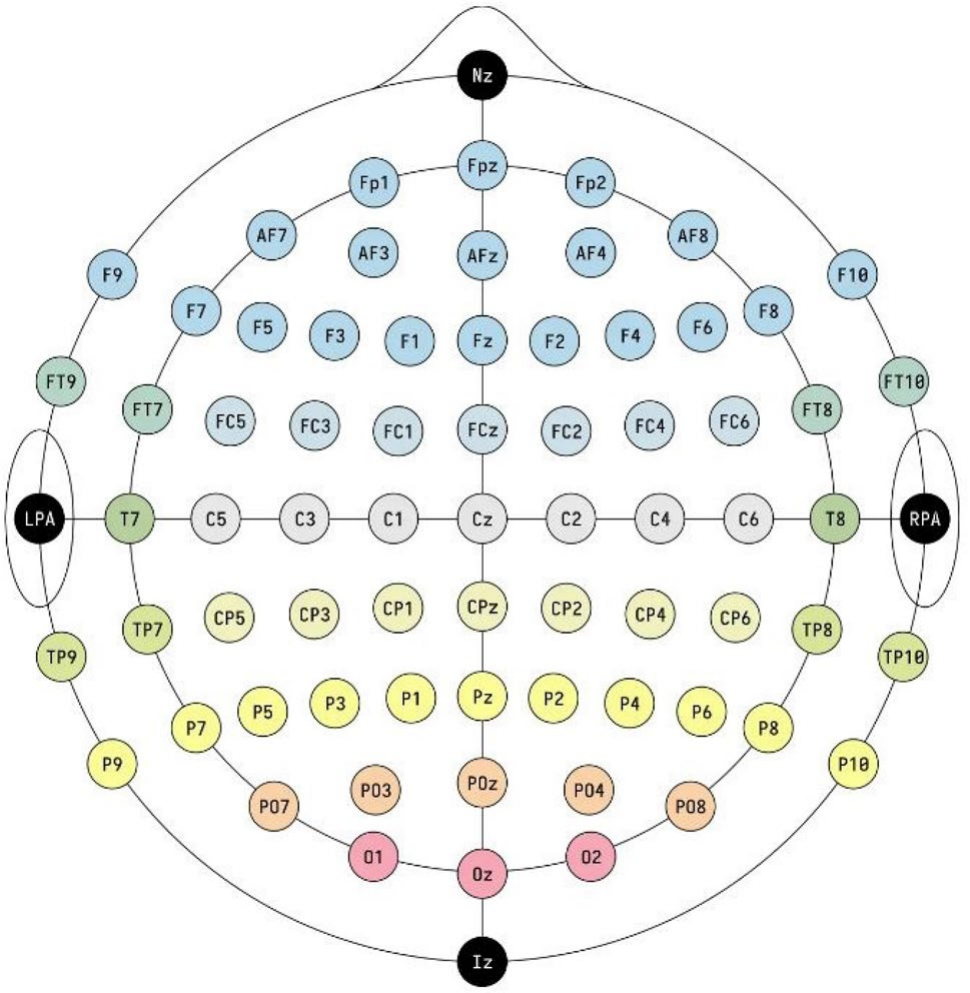
Using modified combinatorial nomenclature, 21 EEG electrode placements based on the 10–20 system were identified.

Assessing and measuring the effectiveness of proposed models involved using patient data obtained from the CHB-MIT database. Assessing and measuring efficacy of obtained data was done using proposed models. The dataset contains long-term EEG scalp readings collected from 23 paediatric patients suffering from intractable seizures and was captured by Boston Children’s Hospital^22^. Using the modified combinatorial nomenclature, the International 10–20 electrode positioning system specifies the names of 21 electrodes that collect 3 channels of EEG signals. The naming convention is presented in Fig. 2. Using data from the Children’s Hospital Boston-Massachusetts Institute of Technology database, this study utilized a sampling rate of 256 Hz that underwent filtering via bandpass method between frequencies ranging from 0 to 128 Hz in order to test how effective our model proposals were. The database contains long term electroencephalogram recordings from the scalps of 23 young patients suffering from seizures that cannot be controlled. The recordings were taken at Boston Children’s Hospital. The electroencephalogram readings were captured using 21 electrodes placed on the patients’ heads following the International 10–20 system, a standardized method to place the electrodes. The electrodes are labelled using specific names as shown in Fig. 2. The electroencephalogram signals were sampled at 256 Hertz and filtered between 0 and 128 Hertz. 16 of the 23 paediatric patients were chosen to assess the classification models. Table 1 has more details on the selected patients^23^. Chb16’s seizures lasted less than 10 s so none were considered for testing. Chb12 and Chb13’s seizures were left out due to changes in channel names and electrode placements. Four patients aged 16 and up (Chb04, Chb15, Chb18, and Chb19) were excluded since the focus is on detecting seizures in young children.

**Table 1:** The selected patients’ seizure data.

Patient ID	Sex-age	Seizure count	Duration of seizure
Chb01	Male-11	8	439
Chb02	Male-12	3	181
Chb03	Female-12	6	399
Chb05	Female-10	6	560
Chb06	Male-14	9	156
Chb07	Male-8	4	319
Chb08	Female-9	5	917
Chb09	Female-6.5	5	279
Chb10	Female-7	6	444
Chb12	Female-12	4	801
Chb13	Female-10	7	172
Chb16	Male-5	4	298
Chb19	Female-9.5	9	299
Chb21	Female-11.5	5	201
Chb22	Female-13	3	208
Chb23	Female-4	8	434
Total		92	6107

Typically, epileptic patients experience fewer seizures which last much shorter compared to periods of seizure-free. There is often an imbalance between the count of seizure EEG data and non-seizure data segments. To address bias when training classification models where models lead to favour the class where the most segments, interictal segments equal the count of ictal segments to form the last dataset. Previous studies have down sampled the original interictal dataset to achieve this balance^24^. For testing the proposed models, 1, 2, and 4 s stipulation were used for non-overlapping EEG segments. The representation of a single EEG segment is a matrix with a dimension of ($$I\times J)$$, here *I,* is the length of sequence = 256 × segment, *J* is the channel count. Let us considered, a 512 × 23 matrix represents one 2-s segment. The EEG dataset is then created by combining all of the ictal and interictal segments into a single matrix with the dimensions (2MN × x23), where M is the total number of ictal or interictal segments and N is the same as previously described. To prepare the EEG dataset prior the training phase, all segments merged undergo the pre-processing through normalization of z-score for all channel to guarantee that all values are standardized by holding a zero-mean ($${z}_{m})$$ and unit standard deviation $$({s}_{d})$$ employing the following Eq. (1),

1 $$y=\frac{y-{z}_{m}}{{s}_{d}}.$$

The entire dataset values are then scaled together to the 0 to 1 range using Min–Max scaling. This ensures that the original and reconstructed segments have similar value ranges. Finally, an extra column is added to the segment’s channels dimension to make it more suitable for the autoencoder model. Publicly available datasets were examined in this study. This information can be found here: https://physionet.org/content/chbmit/1.0.0/.

### Methods

**Figure 3:**
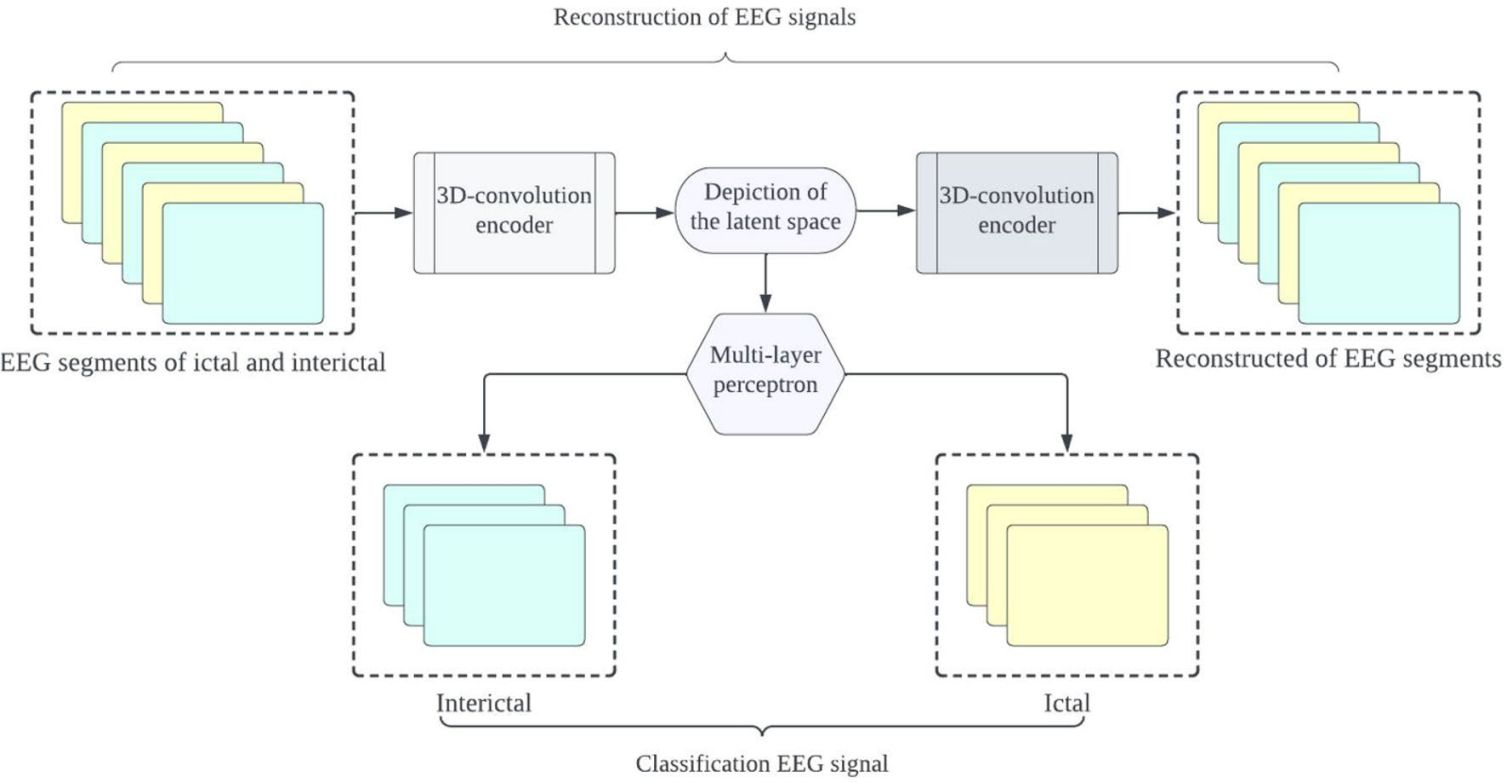
3D-DCAE + MLP architecture of seizure detection.

**Figure 4:**
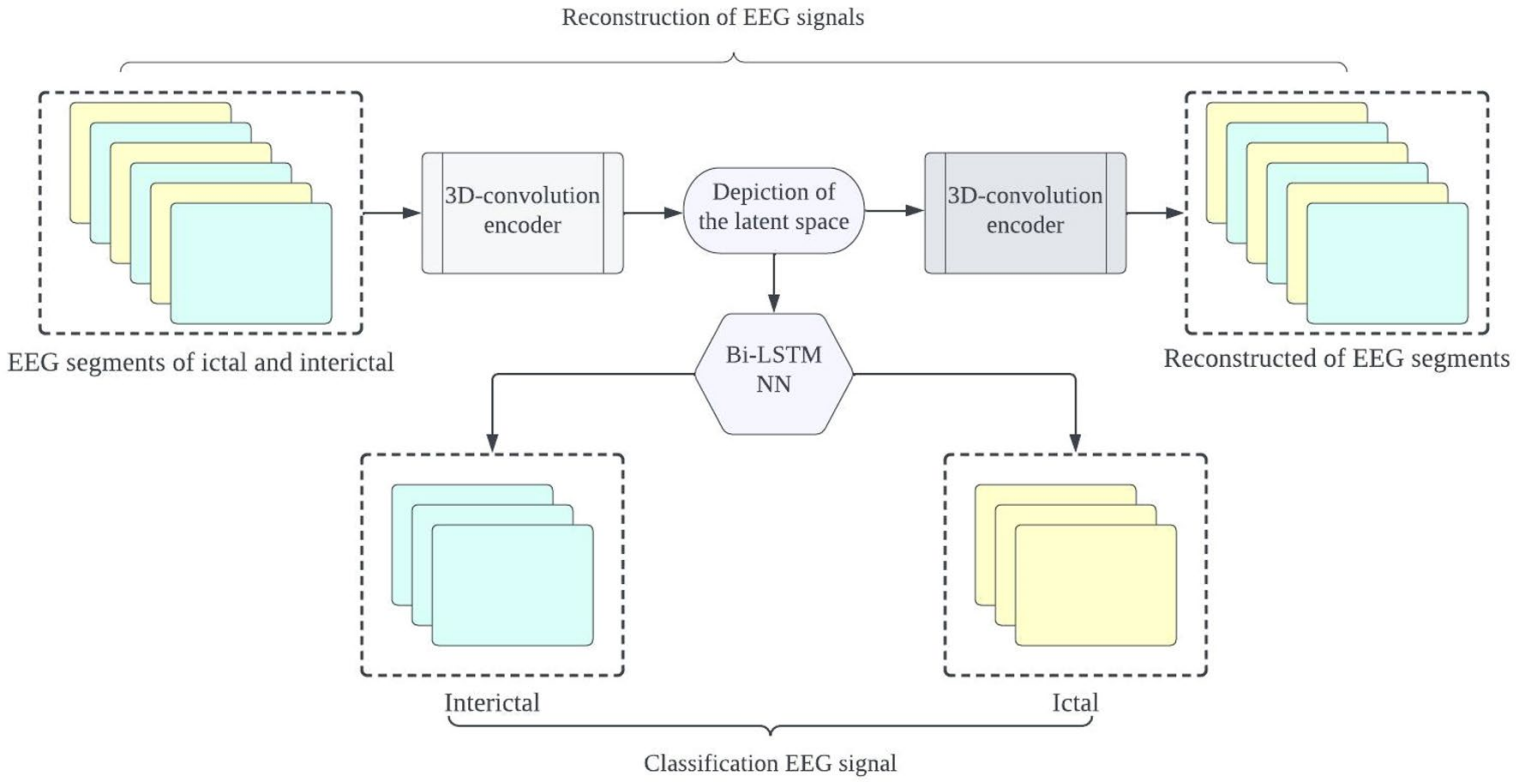
3D-DCAE + Bi-LSTM architecture of seizure detection.

**Figure 5:**
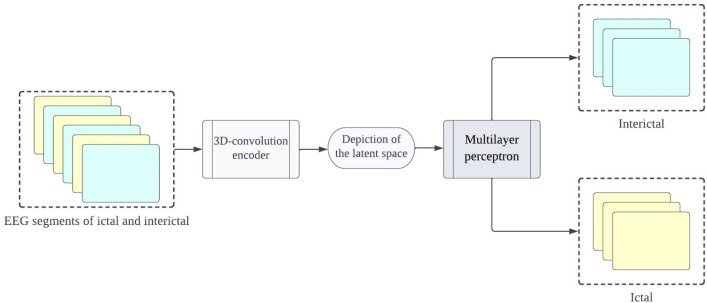
3D neural network with multilayer perceptron.

**Figure 6:**
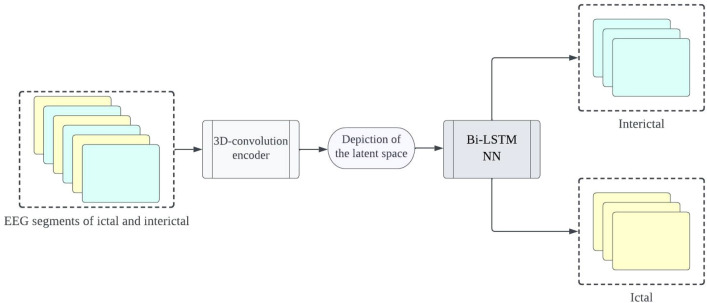
3D neural network with Bi-LSTM neural network.

The proposed framework aims to develop precise and dependable deep learning models for detecting epileptic seizures. The differentiation of two classes of brain states into interictal and ictal is what enables this to be achieved. The powerful features learned by the proposed models contribute to attaining high classification accuracy for minimally pre-processed EEG signals. Our focus is on eliminating the requirement for manual feature extraction by deploying auto-encoders (AEs) in place of complex and time-intensive methods. This will result in faster, simpler, and highly effective systems. A combination of an encoder and decoder make up the AE neural network. The compression of input information (EEG signals) into a lower dimension is done by the encoder, while the decoder decompresses it to recreate the original signal. The network is trained to minimize the loss between the original and reconstructed inputs for AE-based compression. Proposed 3D-DCAE-based models can learn inherent signal features from labelled EEG segments during supervised training. The incorporation of both an encoder producing latent representations and a multilayer perceptron network for classification enables the 3D-DCAE to accurately classify data. Figure 3 portrays the primary proposed model that employs a 3D-DCAE. Also passed into a multilayer perceptron network is the latent space representation that was extracted by the encoder. The classification task can be completed using the MLP. The proposed model featured in the diagram showcases a 3D-DCAE. The latent space representation of the encoder is fed into a Bi-LSTM recurrent neural network for performing classification Shown in Fig. 4. This model’s performance will be evaluated alongside two others. A two-dimensional deep convolutional network (3D-DCNN) along with an MLP is demonstrated in Fig. 5. Figure 6 displays a Bi-LSTM incorporated 3DDCNN.

#### 3 Dimensional deep neural network AE

For the purpose of processing multidimensional data like images and multi-channel EEG signals with high efficiency—convolutional neural network stand out as a well-suited special class among the feedforward neural networks, and very impressive results have been obtained by employing CNNs in various fields including computer vision and pattern recognition. The excellent representation of various types of data through the hierarchical learning of accurate spatial characteristics is what makes deep learning so powerful. Because they rely on parameter sharing and sparse connections where fully connected MLP networks do not, CNNs conserve much more memory, and to improve upon previous models standard AE models are eschewed in favour of those incorporating both convolutions along with pooling so as to provide various added benefits: thus, resulting into an output much superior by employing the proposed 3D Convolutional Auto-Encoder. To build an autoencoder architecture in the form of a CNN consisting of eight different types (four each) respectively alternated with each other are utilized for its encoder component and to analyze input EEG signal segments using deep learning methods, the convolutional layer acquires knowledge about their spatio-temporal properties while max-pooling reduces dimensional requirements via downsampling. A group of filters (kernels), each consisting of tunable weights called filters or kernels constitutes one convolutional layer that slides over and convolves with input data to produce numerous characteristic maps identical to this count, and how much sliding of the filter window occurs over the input is determined by an adjustable parameter (stride). The dimensions of feature maps are reduced through downsampling by the pooling layer in order to decrease computational complexity, and either ‘latent space representation’ or ‘bottleneck’ can be used to refer to a reduced-dimensionality result from an encoding network. The process of reconstructing an original input from a set of interchangeable instructions demands both 4 convolutional as well as 4 up sampling operations within the decoder subnetwork. All models employ an encoder network that features four convolutional layers with filter sizes of 32 and 64 in alternating order for optimal performance and the framework of decoder neural network comprises of initial three convolutional processes incorporating filters in descending order from 64 to dual instances of 32 and preceding them is last single filtered-layer. The fixed parameters for each convolutional layer include a 3 × 2 kernel size and using the default value for stride, so throughout training to preserve the dimensions of feature maps at constant values each convolutional layer uses an identical padding approach. All convolutional layers use rectified linear unit (ReLU) as their activation function except for the final layer, so this can be seen from Eq. (2). The combination of sparsity property with computational simplicity and immunity to noisy input signals makes it.

2 $$g\left(y\right)=\mathrm{max}\left\{0, y\right\},$$

where *y* is the weighted sum of the inputs and *g(y)* is the ReLU activation function. The sigmoid activation function specified in equation is used in the third and final convolutional layer of the 3D-DCAE. Equation (3) to produce an output in the [0, 1] range.

3 $$x=\frac{1}{1+{e}^{-y}},$$

where *y* is the weighted sum of the inputs and *x* is the output of the activation function. In order to reduce input dimensions, we use max-pooling layers configured with windows sized at (2, 2) throughout all but one phase: The last-layer pooling utilizes larger-sized (2, 3) filters and the task of interpolating both rows and columns in input data is completed via usage by means with an interpolation factor set at (2, 3) in the foremost up-sample location. A constant scalar value is used for subsequent up-sampler locations while the utilization of Batch Normalization (batch norm) techniques in our models accelerates and stabilizes the training process while ensuring top-notch performance.4

The batch normalization transform Eq. (4) is shown. Here, the input vector is ${y}_{i}$, and the mini-batch is *M*_*B*_, and the mean, variance can be ${\gamma }_{{M}_{B}}$ and $\alpha$ respectively. Then $\alpha$ and  are the two jointly learned parameters are used to scale and shift the normalised value, and  is added for numerical stability. The encoder subnetwork’s four convolutional and max-pooling layers are separated by four batch normalization layers.

#### Proposed three-dimensional deep convolution autoencoder with MLP

**Figure 7:**
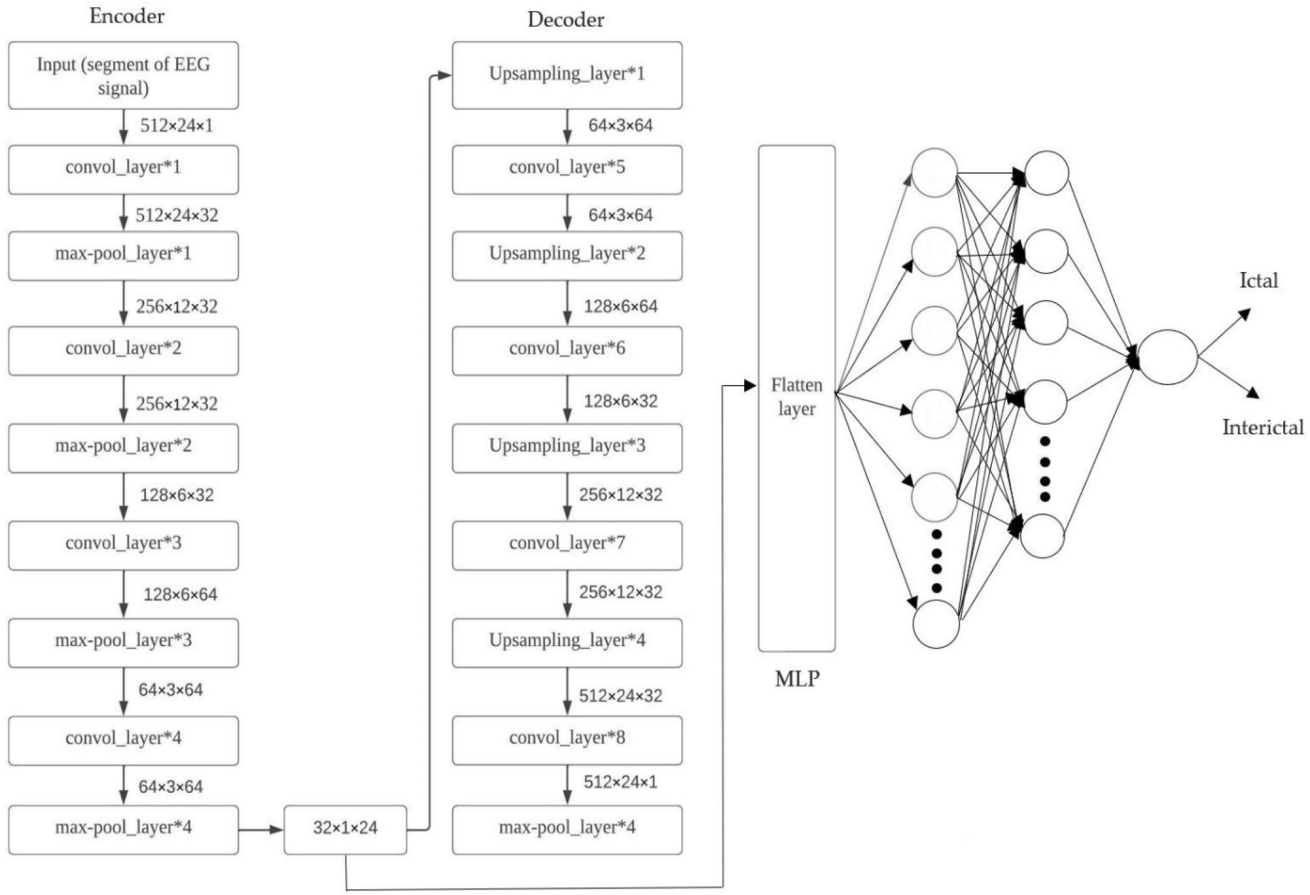
3D-DCAE with MLP architecture.

The initial model presented in Fig. 7 leverages a flatten layer to transform the multi-dimensional latent space representation produced by the decoder subnetwork into a vector shape which is then passed through an MLP-based classifier and in an MLP network architecture there are two hidden fully-connected layers which have a capacity of having up to (or exactly) fifty and thirty-two neurons for each layer respectively. In this model’s both layers make use of the ReLU activation function and the last stage in a neural network’s processing of data is when it produces an ultimate decision through its output layer. The MLP’s use of a sigmoid activation function allows for computation of class probabilities based on input EEG segments.

#### Three-dimensional deep convolution autoencoder with Bi-LSTM network

**Figure 8:**
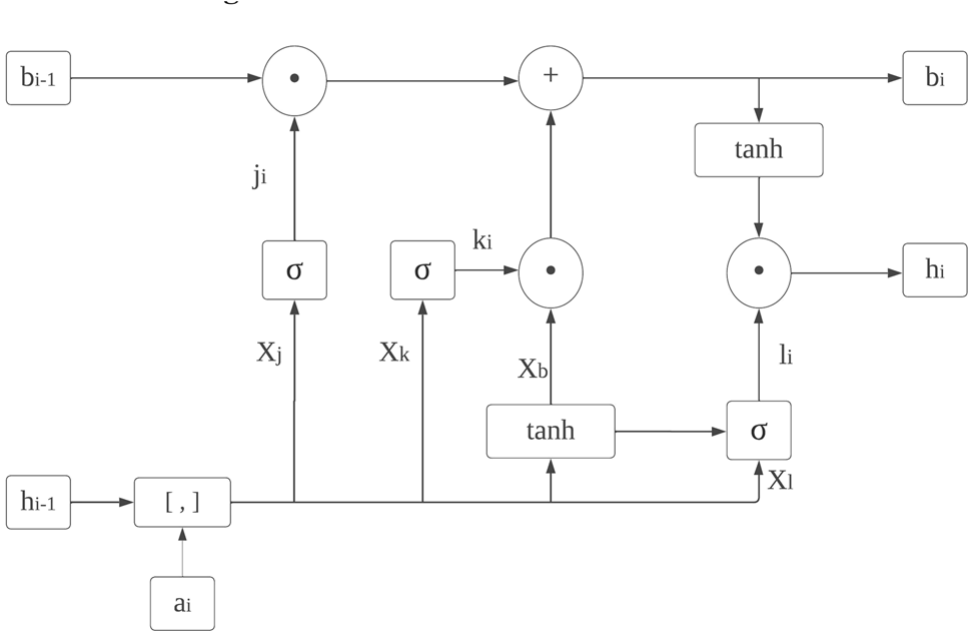
LSTM architecture.

**Figure 9:**
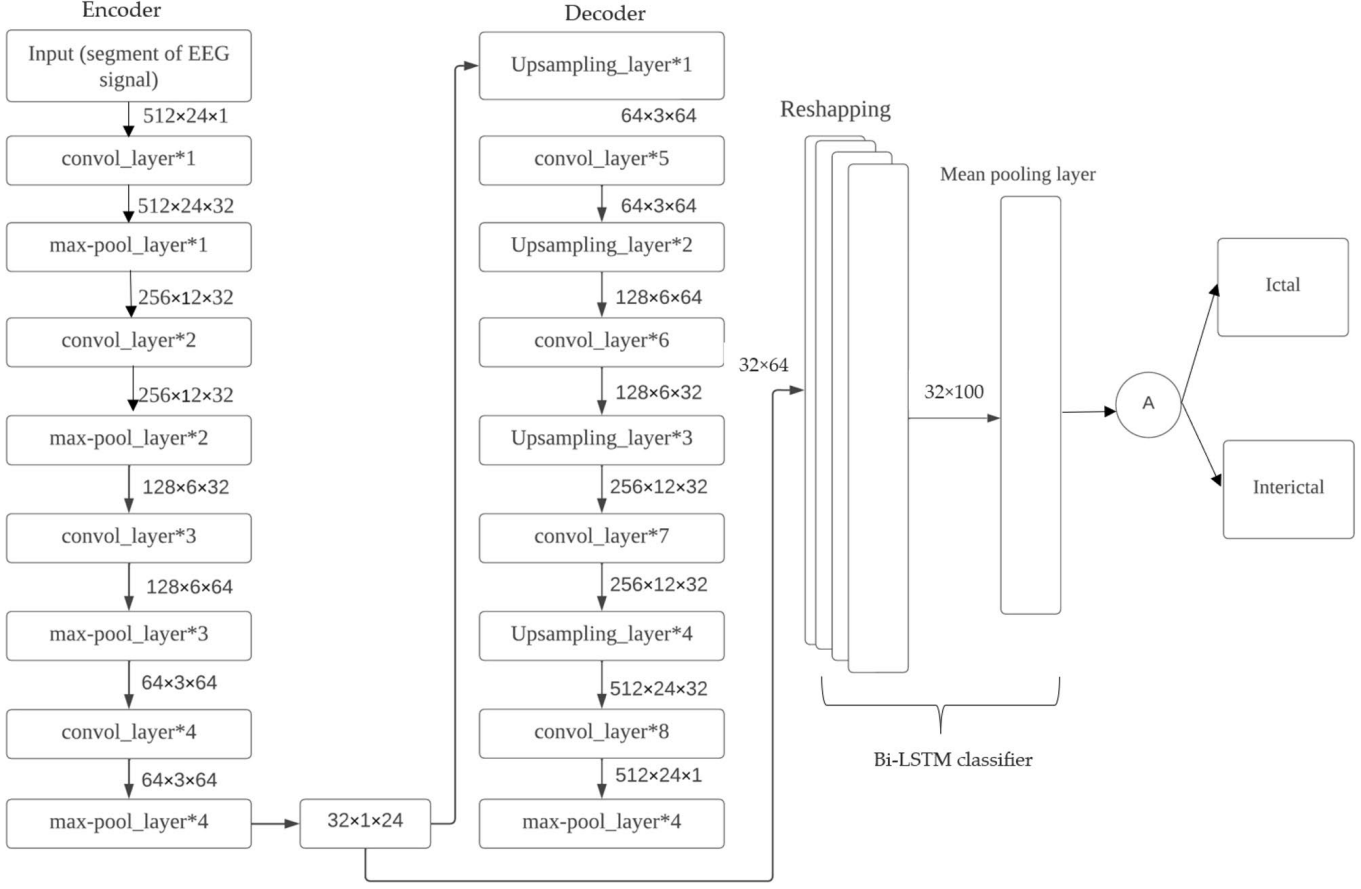
Bi-LSTM recurrent neural network-based classifier for classification according to our second prototype.

LSTM refers to an exclusive design of the recurrent neural network’s structure so to mitigate issues such as gradient explosion/vanishing and information morphing that occur while using vanilla RNNs for training with BPTT; it was developed. By proposing the use of memory cells (units) equipped with three controlling gates for LSTMs networks, it’s possible to maintain gradients values computed by backpropagation during network training and preserve long-term temporal dependencies between inputs, and the configuration for a single LSTM cell is shown in Fig. 8.

5 $${j}_{i}=\sigma \left({X}_{j}\cdot \left[{h}_{i-1},{y}_{i}\right]+{a}_{j}\right),$$

6 $${k}_{i}=\sigma \left({X}_{k}\cdot \left[{h}_{i-1},{y}_{i}\right]+{a}_{k}\right),$$

7 $${l}_{i}=\sigma \left({X}_{l}\cdot \left[{h}_{i-1},{y}_{i}\right]+{a}_{l}\right),$$

8 $${\widetilde{b}}_{i}=\mathrm{tanh}\left({X}_{b}\cdot \left[{h}_{i-1}, {y}_{i}\right]+{a}_{b}\right),$$

9 $${b}_{i}={j}_{i}\odot {b}_{i-1}+{k}_{i}\odot {\widetilde{b}}_{i},$$

10 $${h}_{i}={l}_{i}\odot \mathrm{tanh}\left({b}_{i}\right),$$

where ${y}_{i}$ is the input at time *i* in a sequence Y = (y1; y2; y3;…; y_n_) of *n* time steps. ${h}_{i-1}$ and ${b}_{i-1}$ are the hidden state output and cell state at the previous time step, respectively. ${h}_{i}$ and ${b}_{i}$ are the current hidden state and cell state. ${j}_{i}$, ${k}_{i}$, and ${l}_{i}$ are the forget, input, and output gates. X and a represent the weights and biases matrices and vectors while $\sigma$ is the sigmoid (logistic) function and $\odot$ is the Hadamard product operator. The memory cell starts operation by selecting which information to keep or forget from the previous states using the forget gate ${j}_{i}$. Then, the cell calculates the candidate state ${\widetilde{b}}_{i}$. After that, using the prior cell state ${b}_{i-1}$ and the input gate ${k}_{i}$, the cell decides what further information to write to the current state ${b}_{i}$. Finally, the output gate ${l}_{i}$ calculates how much state information ${h}_{i}$ will be transported to the next time step. Figure 8 is significantly simplified by removing the biases, weight matrices, and operations for multiplication between the concatenated input matrix and the hidden state. The decoded subnetwork’s result from Fig. 9 is fed into a Bi-LSTM recurrent neural network-based classifier for classification according to our second prototype. The architecture of this classification model involves a single-layer Bi-LSTM network that has been designed with two LSTM cells and the structure of a Bi-LSTM architecture is almost identical to that of its counterpart (unidirectional) except it uses dual symmetrically placed Stacked Long Short-Term Memory (LSTM) nodes each receiving input from two opposing other nodes at every instant during processing. The process for classifying an incoming data segment is to combine each block’s output into one array and take its average over all time steps.

**Figure 10:**
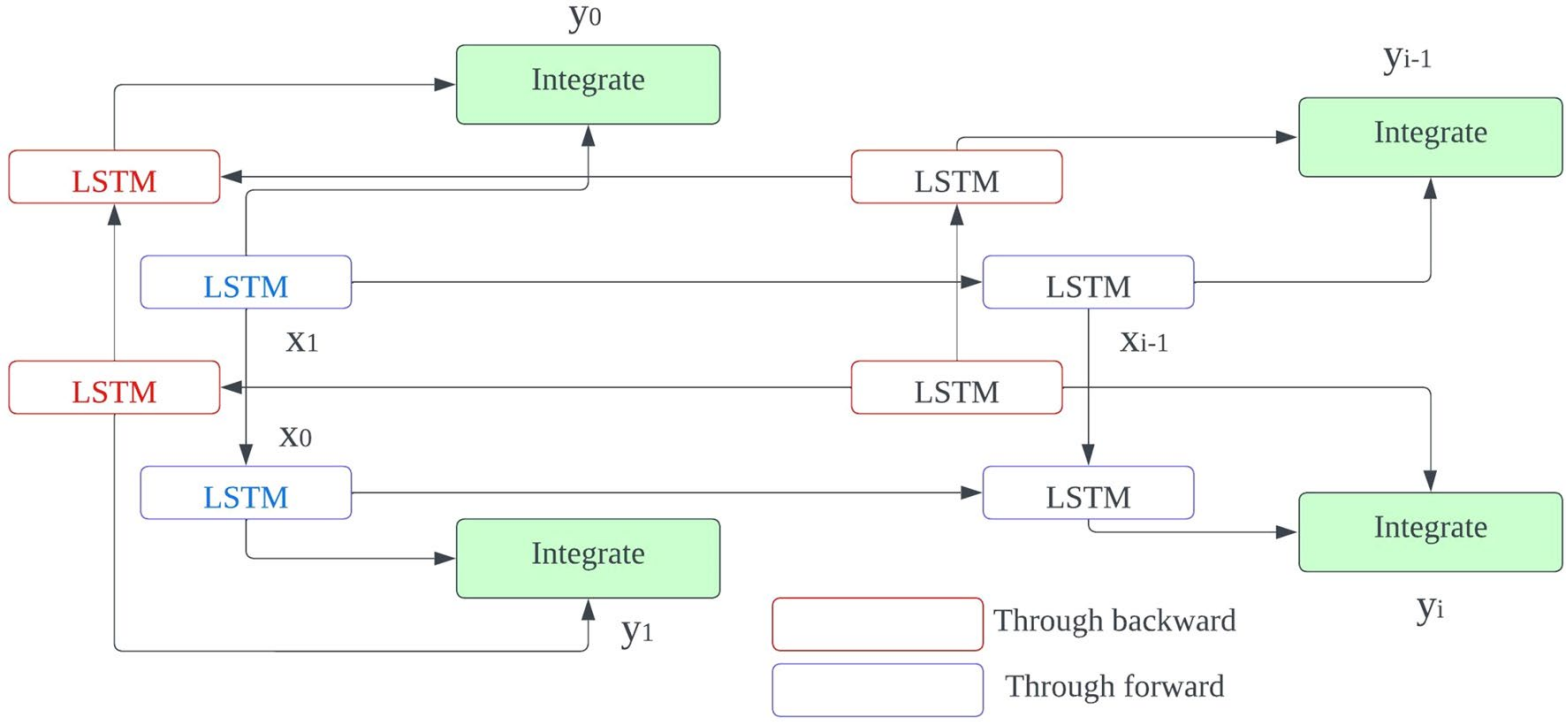
Unrolling of a single-layer Bi-LSTM network over *n* time steps.

Bi-LSTMs are able to improve the accuracy of classifications by considering how each current input relates to those before and after it in time, and Fig. 10 illustrates the unrolling of a single-layer Bi-LSTM network over n time steps. By utilizing dropout regularization method with a value set at 0 and alongside configuring our Bi-LSTM layer as prescribed in our model architecture documentation, namely consisting of fifty neurons (units), we were able to prevent over-fitting. In predicting the EEG segmentation’s category label just as in Model 1, the sigmoid activation feature was employed. Designing the proposed models with a focus on minimizing two losses during network training was necessitated by the simultaneous performance of input reconstruction and classification by the LHCAE, so at the first stage in evaluating performance we use CL labelled classification to assess prediction accuracy. Choosing a loss function was resolved via selecting binary cross entropy from Eq. (11),

11 $${C}_{Loss}=-\frac{1}{M}\sum_{j=0}^{M-1}{x}_{i} \cdot \mathrm{log}\left({\widehat{x}}_{i}\right)+\left(1-{x}_{i}\right) \cdot \mathrm{log}\left(1-\widehat{{x}_{i}}\right),$$

where $\widehat{{x}_{i}}$ is the predicted model output for a single EEG segment, and ${x}_{i}$ is the corresponding actual class label in a training batch equals M.

The mean square error specified by Eq. (12) is used to calculate the second loss, which is the loss of reconstruction (RL_C_) between the original EEG segments and their recreated counterpart decoded by the DCAE.

12 $${RC}_{L}=\frac{1}{M}\sum_{i=0}^{M-1}\frac{1}{qr}\sum_{j=0}^{q-1}\sum_{k=0}^{r-1}{({x}_{jk}-{\widehat{x}}_{jk})}^{2}.$$

Here, the original value ${x}_{jk}$ where the position is indexed by j, k, in a given EEG input and the segment matrix size is (q × r), and the reconstructed value is ${\widehat{x}}_{jk}$ and the total number of segments is denoted by M. Both training processes for deep learning models are quite similar whether it be for models with single or multiple outputs. In similarity to our proposed SDCAE models described earlier in this context—weighting both CL & RL according to Eq. (13) forms TL.

13 $$TL={weight}_{cl}\times CL+{weight}_{rlc}\times {RL}_{c},$$

where the weights can have a value in the regular interval (0, 1), and the proposed model ${weight}_{cl}$ is assigned as 0.5, while ${weight}_{rlc}$ is equal to 1. The backpropagation procedure of loss in both subnetworks we must compute two partial derivatives (gradients): namely, $\frac{\partial TL}{\partial CL}$ and, $\frac{\partial TL}{\partial RLC}$ and weights and biases updates of this model follow the same procedure as typically seen in deep learning that involves calculating all remaining gradients using the chaining rule. Experiments conducted while training the LHCAE included testing different optimizers such as Stochastic Gradient Descent (SGD), root mean square propagation (RMSprop), ADAM and ADADELTA. The selection process took into consideration various model’s performances and resulted in choosing Adam optimizer as the better option compared to others; this included setting its learning rate to 0.0001.

#### Training phase

The effectiveness of our newly-proposed seizure-detection system was measured by comparing it to existing systems using four different types of deep-learning architecture: 3D-DCAE with MLP; 3D-DCAE with Bi-LSTM; 3D-DCNN with MLP; 3D-DCNN with Bi-LSTM. Using diverse performance measurements, we have to test and evaluate 12 different types of models. To guarantee accurate classification of unseen data during model evaluation in the training stage and beyond requires utilizing a tenfold cross-validation methodology with stratification. The methodology adopted for investigating the EEG dataset involves randomization of its contents into ten equally sized subsamples or folds to maintain balance between the two classes—ictal and interticial—within each subset. We have split our dataset into ten parts wherein one-tenth data points are reserved for testing purposes and rest of them are combined together as a training set. The steps involved in cross-validation are performing this action ten times requires each one of the 10-folds to be utilized solely as our test data set. The creation and subsequent training procedure involves conducting iterations where all the models undergo training using batches consisting of only thirty-two samples. The final estimations for various evaluation metrics are derived using the average as well as the standard deviation of the classification outcomes from the 10 iterations.

#### Performance metric evaluation

To assess how well models classify testing sets during ten iterations of tenfold cross-validation, we calculated various widely-used statistical metrics like sensitivity (Se), precision (Pr), accuracy (Acc), specificity (Sp), and F1-score. These performance metric evaluations are expressed as follows,

14 $$Acc=\frac{\alpha +\beta }{\alpha +\beta +\delta +\mu }\times 100,$$

15 $$Se=\frac{\alpha }{\alpha +\mu }\times 100,$$

16 $$Sp=\frac{\beta }{\beta +\delta }\times 100,$$

17 $$F1 score=2\times \frac{Pr\times Se}{Pr+Se}\times 100,$$

18 $$Pr=\frac{\alpha }{\alpha +\delta }\times 100.$$

In Eqs. (14) to (18), the corresponding shorthand notations are as follows: α represents true positive, β denotes true negative, δ signifies false positive, and µ stands for false negative. The notation P stands for positive (ictal) EEG segment count while N denotes negative (interictal). When we talk about TP or TN, we refer to a positive or negative case that is actually correct whereas FP or FN denotes a case that is incorrect in terms of being positive or negative. The meaning of accuracy in this study is based on correctly identifying and classifying EEG segments that belong to a specific state (either interictal or ictal), sensitivity gauges successfully identified seizure-like activities in an electroencephalogram (EEG) whereas specificity assesses accurate classification of non-seizure-related events. Precision tells us how many actual seizures were spotted in a cluster. Finally, the F1-score combines the values of precision and recall in a single metric.

#### Implementation

By using Python programming language alongside additional support software such as the TensorFlow machine-learning library with its Keras Deep Learning API we have developed our model. Because of the diversity of hardware resources and various GPU specifications that were used during training and testing of our suggested models; we have chosen not to utilize computation time as a metric for comparisons. Google Colaboratory is an online environment that runs on Google’s cloud servers and provides us with external resources to develop our models.

## Experimental results and discussion

**Figure 11:**
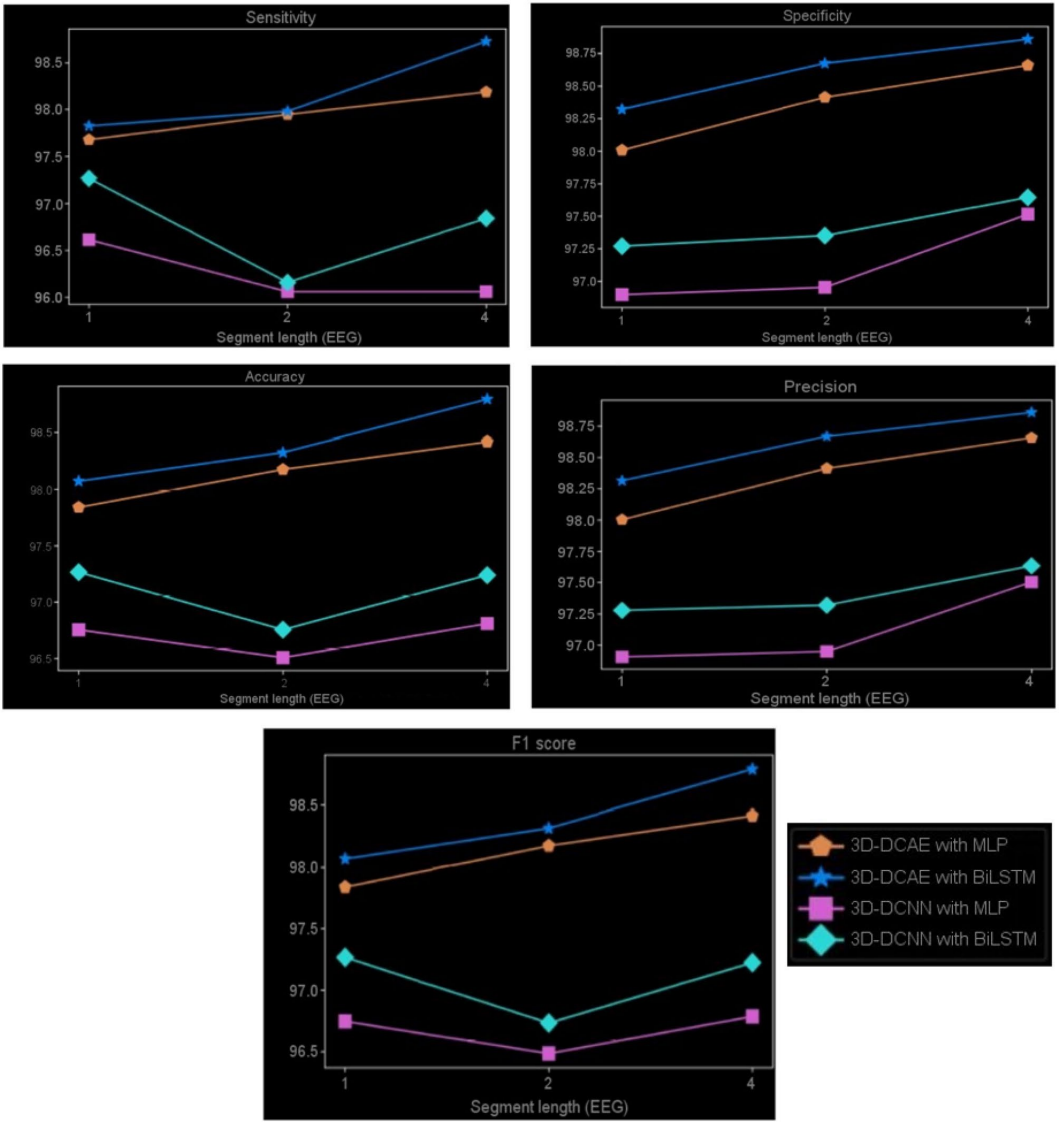
Various EEG segment lengths of classification outcomes.

**Figure 12:**
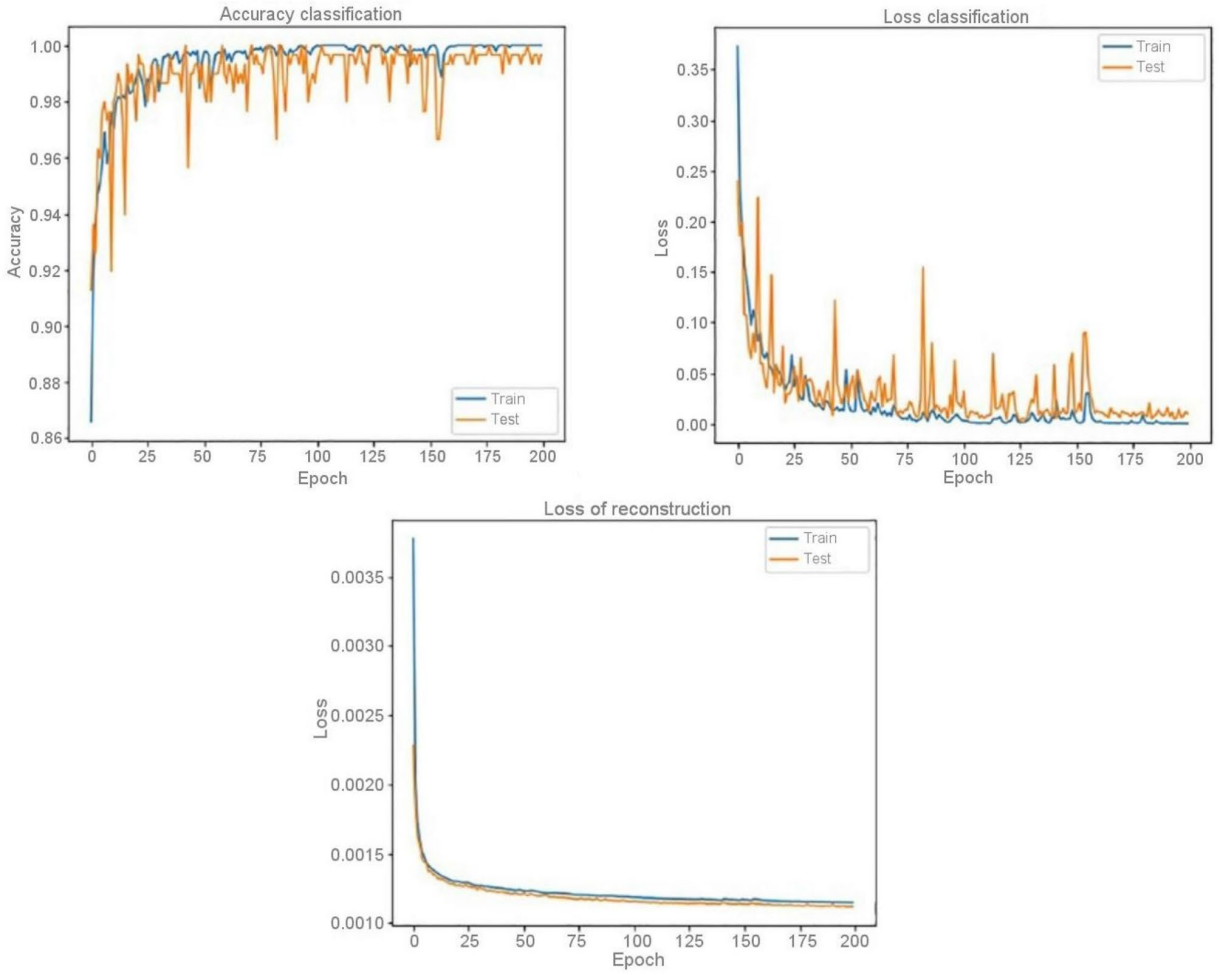
3D-DCAE with Bi-LSTM model accuracy and loss curves while training.

The range of values for five performance metrics, calculated based on classification results drawn from a tenfold cross-validation for EEG segments that were either 1, 2 or 4 s long. These calculations pertain to each of four models. The EEG segments were utilized to derive the performance metrics for the four models. Across the tenfold, Table 2 shows the calculated mean and standard deviation of all metrics. Results indicate that LHCAEs such as 3D-DCEA with MLP & DCEA-Bi LSTM incorporating auto encoders have greatly outperformed competitors without auto encoders across all EEG segmentation length s and assessment measures. Furthermore, Table 2 highlights that the 3D-DCAE model with Bi-LSTM achieved the highest performance among all evaluation metrics at a segment length of 4 s. All other model combinations. It’s worth mentioning that a 4 s EEG segment size is the superior option for optimal classification performance in all LHCAE models. By and large, it can be perceived that all the models which made use of Bi-LSTM for classification realized more favourable consequences as opposed to the models that used MLP-based classifiers employing corresponding EEG segment lengths. EEG signal classification is more effective when using the Bi-LSTM model as compared to MLP-based classifiers. The capability of Bi-LSTM networks to accurately learn temporal patterns from latent space sequences may explain the phenomenon. Figure 11, depicts the different EEG segment lengths of classification results. They perform better than MLP networks. The comparison of standard deviations in evaluation metrics for all models suggests that the LHCAE models tend to demonstrate less dispersion compared to other models. This demonstrates that the LHCAE models’ consistency is maintained during cross-validation iterations. Classification accuracy, classification loss and reconstruction curve data for an achieved model is seen in Fig. 12 which resulted from one of many iterations during a tenfold cross-validation. The employed model consisted of 3D-DCAE and a Bi-LSTM. Both the training and testing datasets were used to accomplish these achievements while in training. The process of first training on a set of data and then evaluating performance with another set—the test data—was applied to this model.

**Table 2:** Results of classification using various EEG segment (length).

Segment (length)	Methods	Se (%)	Sp (%)	Acc (%)	Pr (%)	F score (%)
1 s	3D-DCAE + MLP	97.81 ± 0.79	98.26 ± 0.34	98.23 ± 0.33	98.24 ± 0.32	97.81 ± 0.31
	3D-DCAE + BiLSTM	97.91 ± 0.48	98.57 ± 0.47	98.54 ± 0.46	98.55 ± 0.45	98.12 ± 0.44
	3DCNN + MLP	97.53 ± 1.62	97.15 ± 1.22	97.12 ± 1.21	97.13 ± 1.20	96.7 ± 0.92
	3DCNN + BiLSTM	98.23 ± 0.97	97.52 ± 1.13	97.49 ± 1.12	97.5 ± 1.11	97.07 ± 0.71
2 s	3D-DCAE + MLP	97.91 ± 0.70	98.66 ± 0.94	98.63 ± 0.93	98.64 ± 0.92	98.21 ± 0.51
	3D-DCAE + BiLSTM	97.93 ± 0.48	98.93 ± 0.54	98.9 ± 0.53	98.91 ± 0.52	98.48 ± 0.75
	3D-DCNN + MLP	97.33 ± 1.58	97.2 ± 1.25	97.17 ± 1.22	97.18 ± 1.23	96.75 ± 0.99
	3D-DCNN + BiLSTM	96.23 ± 1.10	97.6 ± 1.58	97.57 ± 1.55	97.58 ± 1.56	97.15 ± 1.11
4 s	3D-DCAE + MLP	99.08 ± 0.79	98.91 ± 0.65	98.88 ± 0.62	98.89 ± 0.63	98.46 ± 0.54
	3D-DCAE + BiLSTM	99.21 ± 0.50	99.11 ± 0.57	99.08 ± 0.54	99.09 ± 0.55	99.16 ± 0.58
	3D-DCNN + MLP	97.10 ± 1.40	97.77 ± 1.24	97.74 ± 1.23	97.75 ± 1.22	97.32 ± 0.52
	3D-DCNN + BiLSTM	98.25 ± 0.93	97.9 ± 1.32	97.87 ± 1.31	97.88 ± 1.30	97.45 ± 1.23

### Performance metric comparison of proposed and state-of-the-art-methods

**Figure 13:**
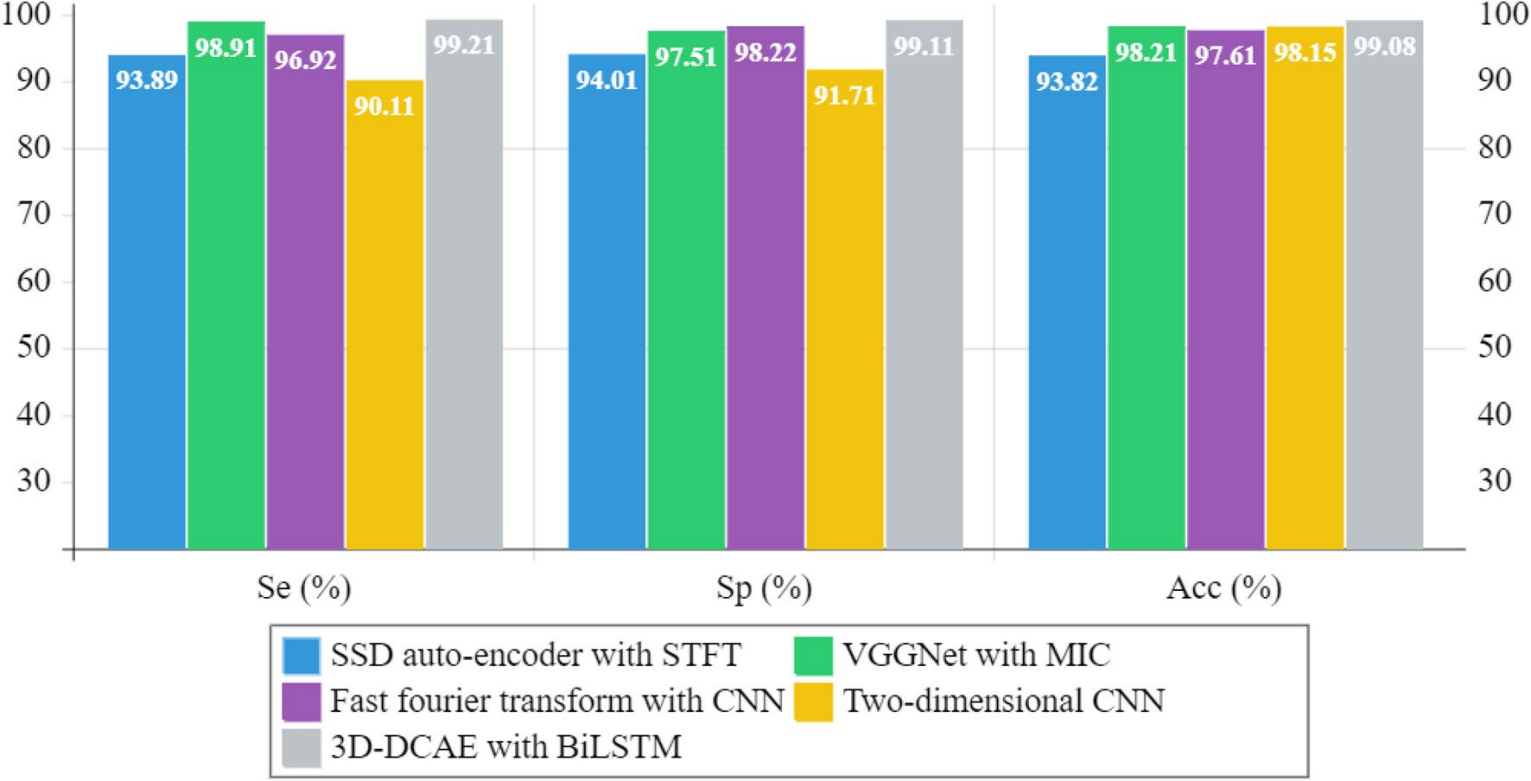
Visualization of metric outcome comparison of existing and proposed model.

Seizure classification algorithm performance is evaluated using different metrics in literature. The challenge arises when trying to differentiate results obtained from various studies due to this factor. Accuracy, sensitivity and specificity are the three widely used metrics that we will use for comparisons in this section. The comparison in Table 3 demonstrates how our highest-performing model stacks up against various advanced methods that use deep neural networks to extract and classify seizures features. Figure 13 depicts the visualization of metric outcome comparison of existing and proposed model with the same dataset. Compared different SSDAEs to assess their feature extraction and classification capabilities. STFT was utilized for pre-processing. They achieved their highest accuracy at 93.82% by randomly selecting the training and testing datasets^24^. Combining feature extraction, global maximal information coefficient (MIC), and visual geometry group network (VGGNet), enables classification of data. Utilizing fivefold cross-validation allowed them to achieve high levels of accuracy (98.21%), sensitivity (98.91%), and specificity (97.51%)^25^. Examined frequency domains using FFT and classified personalized ictal or interictal signal patterns using CNN. An average accuracy, sensitivity, and specificity of 97.62%, 96.92%, and 98.22% respectively were obtained for all patients using a six fold cross-validation approach for evaluation^26^. Spectral and temporal features were extracted by the authors from EEG signals via a 2D-CNN model. A group of training and testing datasets were selected at random for patient-specific classification. Consistently, the cross-patient study produced an accuracy rate, sensitivity rate, and specificity rates as follows: 98.15%, 90.11%, and 91.71% respectively^27^. Some of the most advanced systems were outperformed by our model’s results in the previous comparison. Not one has the accurate statistical analysis required for conducting a test of significance.

**Table 3:** Performance metric outcome comparison of existing and proposed model using the same dataset.

Author	Dataset	Selection of data	Feature extraction	Se (%)	Sp (%)	Acc (%)
^24^	CHB-MIT	Randomized	SSD auto-encoder with STFT	93.89	94.01	93.82
^25^	CHB-MIT	Cross validation—fivefold	VGGNet with MIC	98.91	97.51	98.21
^26^	CHB-MIT	Cross validation—sixfold	Fast Fourier transform with CNN	96.92	98.22	97.61
^27^	Figshare	Randomized	Two-dimensional CNN	90.11	91.71	98.15
^28^	TUH EEG Seizure Corpus	Randomized	Squeeze-and-excitation networks	95.82	96.11	97.80
^29^	University of Bonn	Randomized	Supervised deep convolutional autoencoder	98.72	98.86	98.79
^30^	TUH EEG Seizure Corpus	Randomized	Support vector machines and convolutional neural networks	94.22	94.81	95.0
^31^	CHB-MIT	Six binary classifications	SVM with tunable-Q factor wavelet transform	96.56	97.34	98.78
^32^	CHB-MIT	Three and five-class classification	Superlet transform and VGG19	–	–	94.3
Proposed model	CHB-MIT	Cross validation—tenfold	3D-DCAE with BiLSTM	99.21	99.11	99.08

## Conclusion

A novel approach for detecting seizures in paediatric patients using deep-learning is being proposed. The detection of epileptic seizures is achieved through a novel approach that uses a 3D-LHCAE. This method classifies minimally pre-processed raw multichannel EEG signal recordings. Seizure detection accuracy and efficiency can be improved potentially with this technique. The ability of an AE to learn features automatically while also classifying between ictal and interictal brain state EEG signals is exploited by training it in a labelled hybrid manner. It does this with impressive efficiency. The creation and evaluation of two LHCAE models utilizing BiLSTM and MLP network-based classifiers were based on three different EEG data segments lengths. Two regular deep learning models with the same layers’ structure are compared to both proposed models for their performance. The entire decoder network is taken out with no layers remaining. Training and evaluating the twelve models involve applying a tenfold cross-validation technique. The model that performed the best across all five-evaluation metrics was the LHCAE model incorporating a Bi-LSTM and 4 s EEG segments. The accuracy, sensitivity, specificity, precision and F1 score percentages of this design are: 99.08%, 99.21%, 99.11%, 99.09% and 99.16% respectively. Most of the existing state-of-the-art systems using the same dataset are outperformed by our proposed LHCAE model, which is quite apparent.

## Data Availability

The datasets used during the current study are available from the corresponding author on reasonable request.
